# Site Saturation Mutagenesis Applications on* Candida methylica* Formate Dehydrogenase

**DOI:** 10.1155/2016/4902450

**Published:** 2016-10-25

**Authors:** Gülşah P. Özgün, Emel B. Ordu, H. Esra Tütüncü, Emrah Yelboğa, Richard B. Sessions, Nevin Gül Karagüler

**Affiliations:** ^1^Department of Molecular Biology and Genetics, Faculty of Science and Letters, Istanbul Technical University, Istanbul, Turkey; ^2^Istanbul Technical University Molecular Biology-Biotechnology & Genetics Research Center, 34469 Istanbul, Turkey; ^3^Department of Molecular Biology and Genetics, Faculty of Science and Letters, Yıldız Technical University, Istanbul, Turkey; ^4^Department of Biochemistry, University of Bristol, Bristol, Avon BS8 1TD, UK

## Abstract

In NADH regeneration,* Candida methylica* formate dehydrogenase (*cm*FDH) is a highly significant enzyme in pharmaceutical industry. In this work, site saturation mutagenesis (SSM) which is a combination of both rational design and directed evolution approaches is applied to alter the coenzyme specificity of NAD^+^-dependent* cm*FDH from NAD^+^ to NADP^+^ and increase its thermostability. For this aim, two separate libraries are constructed for screening a change in coenzyme specificity and an increase in thermostability. To alter the coenzyme specificity, in the coenzyme binding domain, positions at 195, 196, and 197 are subjected to two rounds of SSM and screening which enabled the identification of two double mutants D195S/Q197T and D195S/Y196L. These mutants increase the overall catalytic efficiency of NAD^+^ to 5.6 × 10^4^-fold and 5 × 10^4^-fold value, respectively. To increase the thermostability of* cm*FDH, the conserved residue at position 1 in the catalytic domain of* cm*FDH is subjected to SSM. The thermodynamic and kinetic results suggest that 8 mutations on the first residue can be tolerated. Among all mutants, M1L has the best residual activity after incubation at 60°C with 17%. These studies emphasize that SSM is an efficient method for creating “smarter libraries” for improving the properties of* cm*FDH.

## 1. Introduction

Despite enlarging data banks describing novel enzymes, employable enzymes in industry can not meet the increasing demands for enzymatic synthesis. The first reason for this gap is the instability of enzymes extracted from mesophilic organisms which cannot cope with the difficult conditions in industrial synthesis. Second reason is the low availability of extremophilic enzymes which can withstand industrial harsh conditions. Studies in the literature show that less than 1% of biodiversity in the biosphere can be employed industrially [1]. Therefore protein engineering strategies are still critical to improve the properties of industrially applicable enzymes. In addition to traditional protein engineering strategies, for example, directed evolution and rational design, semirational design known as site saturation mutagenesis approach has recently emerged in protein design [2–4]. In order to overcome the time-consuming screening and selection processes of directed evolution, the site saturation which is a combination of both strategies, a new route to obtain the biocatalysts with the desired properties, would be useful. This allows creating a library of mutants containing all possible amino acids (20 naturally occurring amino acids) changes at one or more predetermined target positions in a gene sequence by limiting the enlargement of the library to be screened.

From NADH regeneration point of view the site saturation mutagenesis approach is applied to improve the properties of NAD^+^-dependent* Candida methylica* formate dehydrogenase (*cm*FDH) which is a critical enzyme in pharmaceutical industry [5]. Although there are many dehydrogenases that can carry out the necessary chemistry, the NAD^+^-dependent FDH offers several advantages over any of the other dehydrogenases. It also has been extensively studied as a candidate for developing industrial NADH regeneration. However, the native FDHs have two important disadvantages: lack of thermostability and limited coenzyme specificity. Thus, researchers would like to utilize it in the different reaction systems by redesigning its limited coenzyme specificity and increasing thermostability. Previous studies of this enzyme from different sources have yielded some useful information on both the switching of coenzyme specificity and increasing its thermostability. This is achieved by using rational design approaches [6–14]. However, recently, Andreadeli et al. [15] and Wu et al. [16] used site saturation mutagenesis to alter the coenzyme specificity of* cb*FDH. Asp195Gln/Tyr196His double mutant increased the overall catalytic efficiency of* cb*FDH with NADP^+^. Ordu et al. [7] showed that the substitution of cysteine to the 1st position of* cm*FDH increased the thermostability of the enzyme. Considering this, Met1 is chosen as the target residue to improve the thermal stability of* cm*FDH to apply site saturation mutagenesis.

Here we have applied the site saturation mutagenesis technique both to alter the coenzyme specificity of* cm*FDH from NAD^+^ to NADP^+^ and to increase its thermostability. Two separate libraries were constructed for screening a change in coenzyme specificity and an increase in thermostability.

## 2. Materials and Methods

### 2.1. Construction of Mutant Libraries by Site Saturation Mutagenesis

Degenerate primers containing mutant codon on both forward (5′- CATATGCATGCGGGAGCTCAA**NNK**AAGATCGTTTTAG-3′) and reverse (5′- CTAAAACGATCTT**MNN**TTGAGCTCCCGCATGCATATG-3′) directions were designed to generate the mutant library of Met1 residue for thermostability mutants. Primers which introduced mutations at positions Asp195, Tyr196, and Gln197 were used for constructing the first-generation libraries. Forward primers are** Asp195_F**:5′-CAAAAGAATTATTATACTAC**NNK**TATCAAGCTTTACC-3′,**Tyr196_F**:5′GAATTATTATACTACGAT**NNK**CAAGCTTTACC-3′, and** Gln197_F**:5′-CTACGATTAT**NNK**GCTTTACCAAAAGAAGC-3′ and reverse primers** Asp195_R**:5′-GGTAAAGCTTGATA**MNN**GTAGTATAATAATTCTTTTG-3′,** Tyr196_R**:5′-GGTAAAGCTTG**MNN**ATCGTAGTATAATAATTC-3′, and** Gln197_R**:5′-GCTTCTTTTGGTAAAGC**MNN**ATAATCGTAG-3′ were synthesized using an Applied Biosystems 308A DNA synthesizer. The primers which introduced mutations at positions Tyr196 and Gln197 were used for constructing the second-generation libraries of the Asp195Ser mutant. Forward primers** Asp195Ser/Tyr196_F**:5′-GAATTATTATACTACAGT**NNK**CAAGCTTTACC-3′ and** Asp195Ser/Gln197_F**:5′-CTACAGTTAT**NNK**GCTTTACCAAAAGAAGC-3′ and reverse primers** Asp195Ser/Tyr196_R**:5′-GGTAAAGCTTG**MNN**ACTGTAGTATAATAATTC-3′ and** Asp195Ser/Gln197_R**:5′-GCTTCTTTTGGTAAAGC**MNN**ATAACTGTAG-3′ were synthesized for the alteration of coenzyme (NAD(P)^+^) specificity. Base changes are in boldface. N is A, T, G, or C; K is G or T; M is A or C. Double stranded plasmid DNA containing native* cm*FDH gene was isolated and used as template DNA for PCR studies. Isolated double stranded plasmid DNA containing Asp195Ser mutant* cm*FDH gene was used as template DNA for the second-generation library for the coenzyme specificity studies. The PCR (at 94°C for 2 min initial denaturation, 95°C for 2 min, 50°C for 2 min, and 72°C for 8 min, 30 cycles, and at 72°C for 10 min final extension) was carried out to obtain linear plasmid of* cm*FDH gene with the desired change on it, in the presence of 50 ng template DNA, 0.3 mM of each dNTP, 0.3 pmol of each primer, and 0.02 units of Pfu®* Taq* polymerase. PCR products were digested with 10 units of* Dpn*I (Roche) endonuclease restriction enzyme by incubation at 37°C for 4 hours to eliminate original* cm*FDH genes. Mutant PCR products (Met1 mutants and first-generation (Asp195, Tyr196, and Gln197) and second-generation (Asp195S/Tyr196 and Asp195S/Gln197) mutants) were transformed into* E. coli* BL21 electrocompetent cells (New England Biolabs). To establish mutant library, transformed colonies were grown in 96-well culture plates containing 100 *μ*l Invitrogen MagicMedia* E. coli* expression medium and 100 *µ*g/ml ampicillin by incubation at 37°C for 18 hours. After the growth period, cell cultures containing 20% glycerol in stock plates were stored at −80°C.

Colorimetric assay was applied to monitor NAD(P)H production indirectly, for selecting positive mutants. Colorimetric assay is based on the reduction of nitroblue tetrazolium (NBT) to soluble formazan in the presence of phenazine methosulfate (PMS) which reacts with the NAD(P)H produced by dehydrogenases [17]. For this purpose, cells were grown in 96-deep-well culture plates containing 1500 *μ*l Invitrogen MagicMedia* E. coli* expression medium and 100 *µ*g/ml ampicillin by incubation at 37°C for 22 hours. Cultures were centrifuged at 4000 ×g for 20 min. Pellets were resuspended by using 50 *μ*l “BugBuster” (Novagen) lysis buffer and plates were incubated by shaking at room temperature for 20 min to digest cell and obtain enzymes. After this incubation period cells were centrifuged at 4000 ×g again to remove cell debris. Cleared lysates were taken in new 96-well plates for use in colorimetric screening assay.

Colorimetric assay was repeated for 3 times for all mutants in 200 *μ*l reaction medium containing 20 *μ*l lysate, 20 *μ*l (10 mM) NADP^+^ or NAD^+^, 20 *μ*l (200 mM) formate, and 140 *μ*l reaction solution (50 mM Tris-HCl containing % 0.13 gelatin, pH = 8.0, 300 *μ*M NBT, and 30 *μ*M PMS). Lysate was added as last component to start reaction and increasing blue-purple color was followed at 580 nm to measure activity, indirectly. To determine the thermostability of mutant colonies this assay was performed before and after heat treatment at 55°C for 10 min. After the comparison of mutants to controls, the most promising mutant colonies were selected for further characterization studies.

### 2.2. Purification of Mutant* cm*FDHs

pQE-2 expression vector containing His-tagged FDH gene was transformed into* JM*105 cells for overexpression of NAD^+^-dependent FDH protein. The same protocol [18] was used to purify wild type and mutant* cm*FDH proteins from* E. coli* overexpressing clones. SDS-PAGE and Coomassie Brilliant Blue staining showed the proteins to be >95% pure after purification.

### 2.3. Steady-State Kinetics

The steady-state kinetic experiments were performed at room temperature in a reaction mixture containing 20 mM Tris at pH 8, 1 mM NAD^+^, 0–40 mM formate, and 0.4 *μ*M enzyme or 200 mM formate, 0–40 mM NAD(P)^+^, and 40 *μ*M enzyme. The production of NAD(P)H was monitored by following the increase in absorption at 340 nm. Data were analysed using GraFit 5.

### 2.4. Thermal Denaturation

The thermostability of wild type and catalytically active mutant proteins was determined by measuring the initial rate of NADH production at 340 nm, after incubation of protein samples for 20 min at a series of different temperatures (30, 35, 40, 45, 50, 55, 60, 65, and 70°C). The assay conditions were 0.4 *μ*M enzyme, 20 mM TRIS pH 8 containing 0.2 M formate, and 1 mM NAD^+^.

## 3. Results and Discussion

Through the site saturation mutagenesis, it is possible to create a library of mutants containing all possible combinations of 20 different amino acids at one or more predetermined positions in a gene sequence. In combination with high-throughput screening methods, previously site saturation mutagenesis has successfully been employed to improve several enzymatic properties of FDH and other enzymes [2–4, 15, 16].

**Figure 1:**
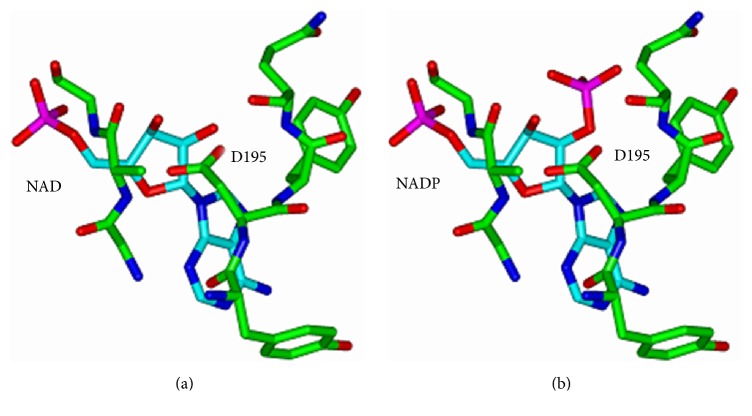
Homology modelling of* cm*FDH based on* Pseudomonas *sp. 101 FDH without mutation in the coenzyme binding domain. (a) Coenzyme is NAD^+^. (b) Coenzyme is NADP^**+**^.

Since there is no crystal structure of* cm*FDH, a homology model of* cm*FDH was constructed by using standard methods based on the crystal structure of* Candida boidinii* FDH (*cb*FDH) [19, 20]. The primary sequence of this protein shown in Figure 1 is 97% identical with that of* cm*FDH. The residues in the coenzyme binding domain and in the catalytic domain which are responsible for the coenzyme specificity and the thermostability are determined by using Insight II (Accelrys) program.

### 3.1. Coenzyme Specificity

In previous experiments we have also achieved* cm*FDH to use NADP^+^ by the single mutation Asp195Ser [11]. However, this mutant binds NADP^+^ weakly. As it is reported in Andreadeli et al. [15] the mutation on Asp 195 and subsequent mutation at 196 generate a few mutant FDH enzymes with NADP^+^ specificity. More recently, Wu et al. revealed that site saturation mutagenesis application on residues Asp195, Tyr196, and Gln197 of* cb*FDH produces more mutants with significant NADP^+^ specificity, which indicate the critical roles of these residues in determining the enzyme's cofactor specificity [16]. In this work, we explore further mutations in the coenzyme binding domain to improve *K*_*m*_ of* cm*FDH for NADP^+^. The single mutations at Asp195, Tyr196, and Gln197 in the coenzyme binding domain are introduced by using the site saturation mutagenesis. The library of coenzyme specificity mutants is screened for the activity towards NAD^+^ and NADP^+^in the presence of several concentrations of sodium formate. The recombinant wild type* cm*FDH is used as the control in each screening plate. 27 colonies showed activity with NADP^+^ from nearly 400 screened colonies in the first screening by using the colorimetric assay. Detailed kinetic measurements are carried out with 5 cultured colonies out of these 27 colonies mentioned above. The activities of recombinant wild type and mutant* cm*FDH enzymes are assayed in the same way mentioned in Section 2.3.

Among them, in the presence of NADP^+^ single mutants Asp195Ser and Gln197Val exhibit the highest catalytic efficiency (*k*_cat_/*K*_*m*_), being 8 M^−1^s^−1^ and 18 M^−1^s^−1^, respectively. Asp195Ser, described as a promising mutant for NADP^+^ specificity by rational design before [11], is obtained from the first engineering round site saturation mutagenesis at position 195. On the other hand, Asp195Ser is also represented as NADP^+^ specificity mutant by Andreadeli et al. [15]. These results confirm that the serine is a suitable residue at this position for NADP^+^ binding. Both the Asp195Ser mutant* cm*FDH enzyme and the wild type* cm*FDH enzyme show similar catalytic efficiency with NAD^+^ (0.22 and 0.32 × 10^3^ M^−1^ s^−1^, resp.). The Asp195Ser is at least 8000-fold more efficient at turning over NADP^+^ than the wild type. The other important mutant Gln197Val obtained from first round of site saturation mutagenesis is different from mutants described before in the literature for switching coenzyme specificity of FDH from NAD^+^ to NADP^+^ [15, 16]. The Gln197Val mutant shows that more hydrophobic residues at position 197 have a greater affinity for NADP^+^. Gln197Val mutant* cm*FDH enzyme shows >18000-fold higher catalytic efficiency towards NADP^+^ than wild type enzyme. However, the catalytic efficiency towards NAD^+^ is (0.06 × 10^3^ M^−1^ s^−1^) 5-fold less than the wild type. The ratios of the catalytic efficiencies (*k*_cat_/*K*_*m*_)^NADP+^/(*k*_cat_/*K*_*m*_)^NAD+^ for single mutant proteins Asp195Ser and Gln197Val are 0.036 and 0.3, respectively.

Mutation on residue 195, which has a proven role in the substrate specificity of FDH, is selected from the first-generation mutant library as a template to construct the second-generation mutant library. In other site saturation mutagenesis studies for changing coenzyme specificity of FDH, Andreadeli et al. [15] engineered the amino acid residue at position 196 using Asp195Gln as a parental sequence. Wu et al. [16] used both Asp195Ser and Asp195Gln as parental sequences and the amino acid residue at position 196 is subjected to second-round mutagenesis. In our study, second-generation mutations are introduced into residues at positions 196 and 197 unlike previous site saturation studies for changing substrate specificity. The library is screened for the activity towards NAD(P)^+^ and 6 double mutants are selected as promising NADP^+^ specific FDH mutants and all cultured for characterization of *K*_*m*_ and *k*_cat_ values for NAD^+^, NADP^+^, and formate (Table 1).

In the second generation, the Michaelis constant (*K*_*m*_) value of mutant Asp195Ser/Tyr196Leu with NAD^+^ is 0.7 mM which is very close to that of wild type* cm*FDH with NAD^+^ (0.62 mM). *K*_*m*_ value of these enzymes in the presence of NADP^+^ is 2 mM which is the best affinity among the 6 double mutants. The catalytic constant (*k*_cat_) of this mutant for NADP^+^ is 2.5-fold higher than that of wild type enzyme whereas the catalytic constant for NAD^+^is about the same as the wild type. This mutant has 0.1 ± 0.02 s^−1^ kcat value whereas catalytic constant of wild type enzyme for NADP^+^ is not determined. Another advantage of this double mutant is its catalytic efficiency with NAD^+^ (0.26 × 10^3^ M^−1^ s^−1^) which is close to the wild type catalytic efficiency of NAD^+^ (Table 1). The catalytic efficiency of this enzyme in the presence of NADP^+^ is 5 × 10^4^-fold higher than the wild type enzyme.

The Asp195Ser/Gln197Thr double mutant displays the highest catalytic constant for NADP^+^ (0.26 s^−1^) and *K*_*m*_ value of NAD^+^ is 0.22 mM. The Asp195Ser/Tyr196Ala double mutant produces the highest affinity (0.13 mM) for NAD^+^ which is about 3 times higher than that of wild type* cm*FDH, without changing the catalytic constant of NAD^+^. Compared to wild type* cm*FDH enzyme Asp195Ser/Gln197Thr double mutant increases the overall catalytic efficiency (*k*_cat_/*K*_*m*_) of NADP^+^ to 5.6 × 10^4^ times higher than wild type* cm*FDH enzyme. The affinity for formate of Asp195Ser/Tyr196Leu and Asp195Ser/Gln197Thr enzymes is 21-fold. This is 5-fold higher than the wild type enzyme in the presence of 200 mM NADP^+^.

Results showed that beside hydrophobicity and polarity, regarding the size of the coenzyme binding pocket, conformation of amino acids is an important parameter for NADP^+^ binding. Characterization of single and double mutants obtained from our study gives substantial amount of data for understanding of coenzyme binding specificities of FDH enzyme and the binding of NAD^+^ or NADP^+^. However, engineering of coenzyme specificity towards industrial demands is a challenging area due to its complexity and molecular mechanisms which have not been fully clarified yet.

### 3.2. Thermostability

It can be seen from the previous studies that there are many attempts to change methionine into Gln, Leu, Ile, Phe, Val, Ala or noncoded amino acid substitution at several positions of different proteins by using site directed mutagenesis [21–23]. Ordu et al. [7] showed that the replacement of methionine to cysteine, as in the case of substitution on residue 1, increases the catalytic efficiency of the protein and leads to an improvement in the thermostability of the secondary structure of* cm*FDH. This is why we have applied the site saturation mutagenesis to the first residue, methionine, of the* cm*FDH to increase its thermostability and to observe the amino acid substitution tolerance of the first residue.

The library of thermostability mutants is screened by tracing the increase of blue-purple color at 580 nm to measure activity, indirectly. To determine the thermostability of mutant colonies, this assay is performed before and after the heat treatment at 55°C for 10 min. The empty MagicMedia* E. coli* expression medium, cells without the* cm*FDH gene, and the native* cm*FDH protein are all used as controls. After comparing mutants with controls, the most promising 8 mutants from 200 screened colonies are selected for further characterization studies (Table 2).

The first residue of* cm*FDH corresponds to the 15th residue of recombinant* 6x*His-tagged* cm*FDH (MKH_6_HMHAGAQ**M**KIVL…). Therefore, mutation on the start codon of the original* fdh* gene does not inhibit the expression of enzyme. As shown by the results of 8 mutations at this position, these alterations are tolerated. If *K*_*m*_ values are compared individually, except for Met1Arg and Met1Leu, all mutants have better affinity for the formate compared to native* cm*FDH. On the other hand, while Met1Leu has a higher *K*_*m*_ value (6.3 mM) than the native* cm*FDH for formate (4.75 mM), it has better *k*_cat_ value (1.2 s^−1^) than that of native* cm*FDH (1.1 s^−1^) and *T*_0.5_ value of this mutant is almost the same as native* cm*FDH (Table 2). When all mutants are compared with each other in terms of catalytic efficiency, Met1Cys (0.4 s^−1^ mM^−1^) is found to be the best one.

**Figure 2:**
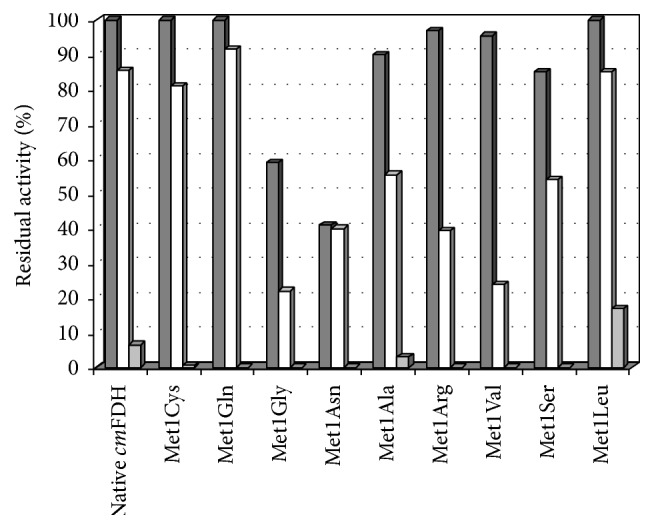
Bar graph presentation of residual activities of native* cm*FDH and site saturation mutants at position 1 after incubation in 50 (dark grey), 55 (white), and 60°C (light grey) for 20 min. Residual activities were expressed relative to activities in room temperature (25°C).

Considering the percentage of residual activity based on thermal inactivation experiments, it is observed that the native* cm*FDH, Met1Cys, Met1Gln, and Met1Leu mutants have almost the same activity after incubation at 50 and 55°C. Among all mutants, Met1Leu has the best residual activity after incubation at 60°C with 17% (Figure 2).

The substitution effect of glutamine probably comes from side chains which have relatively greater polarity than the hydrophobic side chain of methionine. These side chains change the reduction of hydrophobicity and van der Waals interactions [24–26]. Leucine and methionine have hydrophobic amino side chains and the van der Waals volume occupied by leucine is close to methionine. Introduction of the leucine can cause significant steric interferences and substantial movement in several nearby residues [22]. Methionine-to-leucine substitution may give either a stabilizing or destabilizing effect depending on the environment of the residues altered [23]. In this study the stabilizing effect of the Met1Leu mutation comes from increased entropy at room temperature. Compared to native* cm*FDH, higher enthalpy and entropy and lower heat capacity are the notable properties of Met1Leu.

Asparagine and serine have destabilizing effects; arginine, valine, and serine have little effect on stability. The counterpart studies in the literature have shown that methionine substitutions to noncoded amino acids, valine, arginine, and serine generally have minor effects on protein structure. This effect could be either slightly stabilizing or destabilizing depending on the location of the residues [21].

## 4. Conclusion

We applied site saturation mutagenesis to change the properties of* cm*FDH. The cofactor specificity of* cm*FDH was altered from NAD^+^ to NADP^+^by mutating the residues at Asp195, Tyr196, and Gln197. The thermostability of* cm*FDH was also successfully increased by mutating the residue at Met1. These studies showed that site saturation mutagenesis is an efficient method for creating “smarter libraries” for improving the properties of* cm*FDH.

## Figures and Tables

**Table 1:** The kinetic parameters of wild-type *cm*FDH and mutants.

Enzymes	NAD^+^			NADP^+^			(*k*_cat_/*K*_*m*_) NADP^+^/(*k*_cat_/*K*_*m*_) NAD^+^
	*K*_*m*_ (mM)	*k*_cat_ (s^−1^)	*k*_cat_/*K*_*m*_	*K*_*m*_ (mM)	*k*_cat_ (s^−1^)	*k*_cat_/*K*_*m*_	
Native* cm*FDH	0.62 ± 0.3	0.2 ± 0.1	320	ND	ND	<10^−3^	
195Ser	1.1 ± 0.5	0.22 ± 0.1	220	5 ± 1.2	0.04 ± 0.09	8	0.036
197Val	3.3 ± 0.2	0.22 ± 0.1	60	1.4 ± 1.1	0.03 ± 0.05	18	0.3
195Ser/197Val	3.9 ± 2.3	0.16 ± 0.1	40	2.2 ± 1.1	0.01 ± 0.08	4.5	0.113
195Ser/197Thr	0.22 ± 0.03	0.2 ± 0.01	900	4.6 ± 3	0.26 ± 0.1	56	0.06
195Ser/196Leu	0.7 ± 0.2	0.2 ± 0.1	260	2 ± 0.6	0.1 ± 0.02	50	0.2
195Ser/196Ser	2.3 ± 1.8	0.2 ± 0.05	90	8.2 ± 4	0.16 ± 0.05	19	0.2
195Ser/196Ala	0.13 ± 0.1	0.2 ± 0.1	1460	8.5 ± 2.8	0.17 ± 0.1	20	0.014

**Table 2:** Activity and *T*_0.5_ values of site saturation mutants on Met1 position of *cm*FDH.

*cm*FDH Met1 mutants	*K*_*m*_ (mM)	*k*_cat_ (s^−1^)	*k*_cat_/*K*_*m*_ (s^−1^ mM^−1^)	*T*_0.5_
Native* cm*FDH	4.75 ± 0.3	1.1 ± 0.1	0.24	56.7 ± 0.2

Met1Cys	3.3 ± 0.4	1.25 ± 0.005	0.4	58.4 ± 0.2 [7]
Met1QGln	2.9 ± 0.4	0.4 ± 0.002	0.13	54.65 ± 0.2
Met1Gly	2.6 ± 0.3	0.1 ± 0.0005	0.04	53.2 ± 1.3
Met1Asn	2.8 ± 0.3	0.3 ± 0.001	0.1	53.3 ± 1.8
Met1Ala	4.02 ± 0.45	0.4 ± 0.002	0.1	55.6 ± 0.7
Met1Arg	9.8 ± 4.5	0.6 ± 0.01	0.06	55.8 ± 3
Met1Val	3.3 ± 0.2	0.5 ± 0.001	0.16	52.55 ± 0.5
Met1Ser	4.5 ± 0.5	0.9 ± 0.004	0.2	55.1 ± 0.7
Met1Leu	6.3 ± 1	1.2 ± 0.008	0.18	56.6 ± 0.2
